# Complete Genome Sequence of *Exiguobacterium* sp. Strain MH3, Isolated from Rhizosphere of *Lemna minor*

**DOI:** 10.1128/genomeA.01059-13

**Published:** 2013-12-19

**Authors:** Jie Tang, Ying Zhang, Hao Meng, Zhiquan Xue, Jiong Ma

**Affiliations:** School of Environment and Energy, Peking University Shenzhen Graduate School, Shenzhen, Guangdong, China

## Abstract

We report the complete genome sequence of *Exiguobacterium* sp. strain MH3, isolated from the rhizosphere of duckweed. The genome assembly is 3.16 Mb, with a G+C content of 47.24%, and it may provide useful information about plant-microbe interactions and the genetic basis for the tolerance of the strain to various environmental stresses.

## GENOME ANNOUNCEMENT

Members of the genus *Exiguobacterium* are widely distributed in diverse habitats, including plant rhizospheres (1), biofilms (2), freshwater (3), marine waters (4), hot/cold environments (5, 6), and extreme conditions (7, 8). To date, 14 species of the genus have been isolated and characterized. They show great potential in industrial applications, including biotechnology, agriculture, food, and pharmaceutical industries, among others (6, 8, 9). Seven strains have had their genomes sequenced recently (6, 7), but none were isolated from a rhizosphere.

Duckweed, the smallest flowering plant, has been used widely for environmental monitoring and bioremediation and, lately, for bioenergy (10). Employing an ultrasonic method, *Exiguobacterium* sp. strain MH3, a psychrophilic facultative anaerobic bacterium, was isolated from the rhizosphere of a duckweed strain, *Lemna minor*, collected from Shenzhen, China (22°35′5.47″N, 113°57′36.37″E). On the basis of its 16S rRNA sequence, we determined that it is a member of the *Exiguobacterium* genus. MH3 was characterized as Gram-positive, non-spore-forming, having good mobility with flagella, and being able to grow between 4 and 40°C, at pH levels from 5 to 9.5, and with up to 10% NaCl (wt/vol).

Whole-genome shotgun sequencing of *Exiguobacterium* sp. MH3 was performed at the Beijing Genomics Institute (BGI) (Shenzhen, China) using Illumina HiSeq 2000 on a combination of 0.5-kb and 6.5-kb DNA libraries. This generated a total of 5,566,668 filtered paired-end reads, providing 140-fold coverage of the genome. The SOAP*denovo* alignment tool (11) assembled the reads into 1 scaffold, comprising 3 contigs. The gaps within the scaffold were confirmed and closed using long-distance PCR amplification by Sanger sequencing. Finally, a single replicon (total, 3,164,195 bp), with a G+C content of 47.24%, was obtained for the genome of *Exiguobacterium* sp. MH3.

Gene prediction and genome annotation were performed on the RAST server and the NCBI PAPPC (12, 13). tRNA and rRNA sequences were identified using tRNAscan-SE and RNAmmer, respectively (14, 15). In total, 3,203 coding sequences (CDSs) representing 398 predicted SEED subsystem features were predicted, including 2,479 proteins with identified functions. Nine rRNA operons and 60 tRNAs were also annotated. As expected, many stress tolerance-related genes were identified, including putative genes encoding a Pho regulon for high-affinity phosphate uptake (6), carbon starvation, oxidative stress, detoxification, and cold and heat shock proteins. Interestingly, genes related to auxin biosynthesis, siderophores, and iron acquisition and metabolism are also found in the MH3 genome, which may contribute to the promotion of plant growth, a definite benefit to its plant host.

Further analysis of the genome, including functional, molecular, and biochemical studies and plant-microbe relationship analyses, will be used to understand the mechanisms of MH3 for environmental stress tolerance, as well as its possible functions in promoting plant growth and stress resistance. This is the eighth draft genome for the genus *Exiguobacterium* but the only one isolated from a rhizosphere. It will provide a reference for many further phylogenetic, comparative genomic, metagenomic, and functional studies of this widely distributed genus.

### Nucleotide sequence accession number.

This whole-genome shotgun project has been deposited at DDBJ/EMBL/GenBank under the accession no. CP006866.
